# Supplementary material for: The actin cytoskeleton plays multiple roles in structural colour formation in butterfly wing scales
Source: Nat Commun. 2024 May 20;15:4073. doi: 10.1038/s41467-024-48060-3 (PMC11106069; doi:10.1038/s41467-024-48060-3)
Supplement: Supplementary file 4 — Description of Additional Supplementary Files [file 41467_2024_48060_MOESM4_ESM.pdf]

Supplementary Movie 1

Movie of an X-ray nanotomography reconstructed blue, iridescent scale. False coloured, blue.

Supplementary Movie 2

Movie of an X-ray nanotomography reconstructed non-iridescent, black scale. False coloured, black.

Supplementary Movie 3

Animated Z-stack of an actin bundles (green) in an iridescent scale at 44% development (Fig 4A-C). Dashed line indicates the outline of the scale cell and yellow arrows correspond to the points at which finger formation begins on the distal cell edge.
