# Supplementary figures and images for: The actin cytoskeleton plays multiple roles in structural colour formation in butterfly wing scales
Source: Nat Commun. 2024 May 20;15:4073. doi: 10.1038/s41467-024-48060-3 (PMC11106069; doi:10.1038/s41467-024-48060-3)

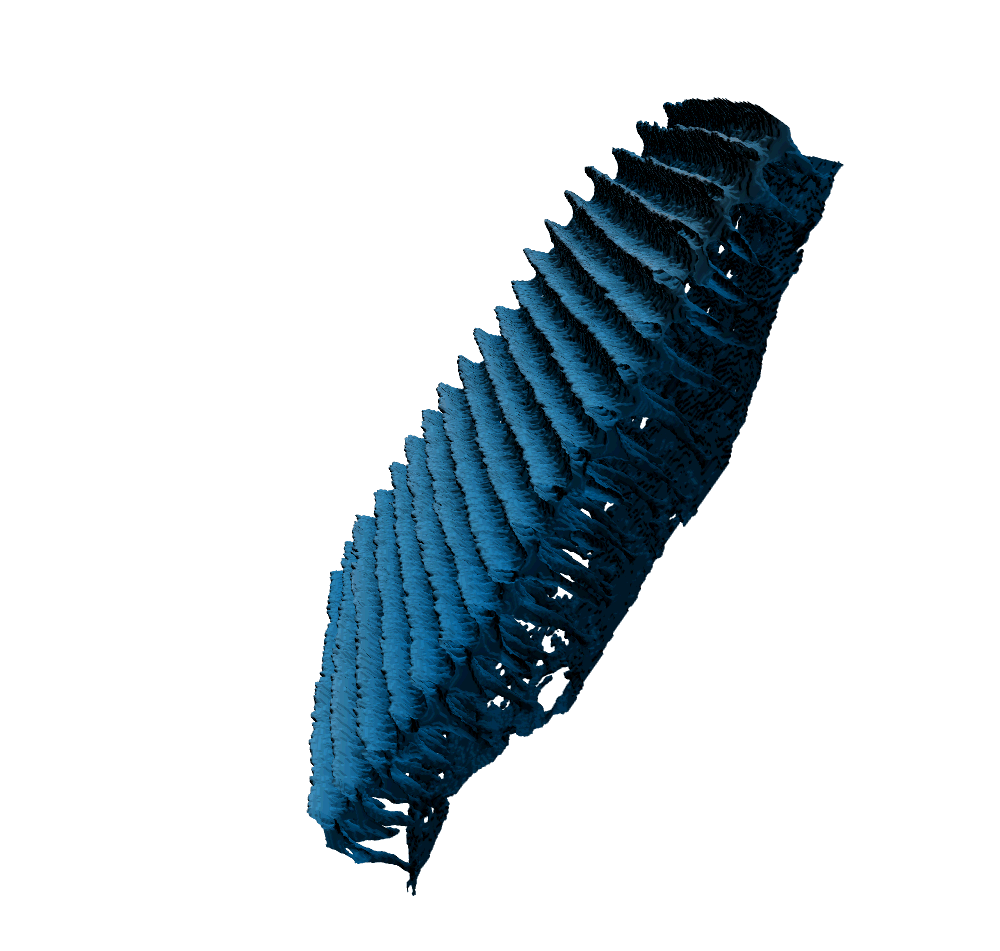

Supplement: Supplementary file 5 — Supplementary Movie 1 [file 41467_2024_48060_MOESM5_ESM.gif]

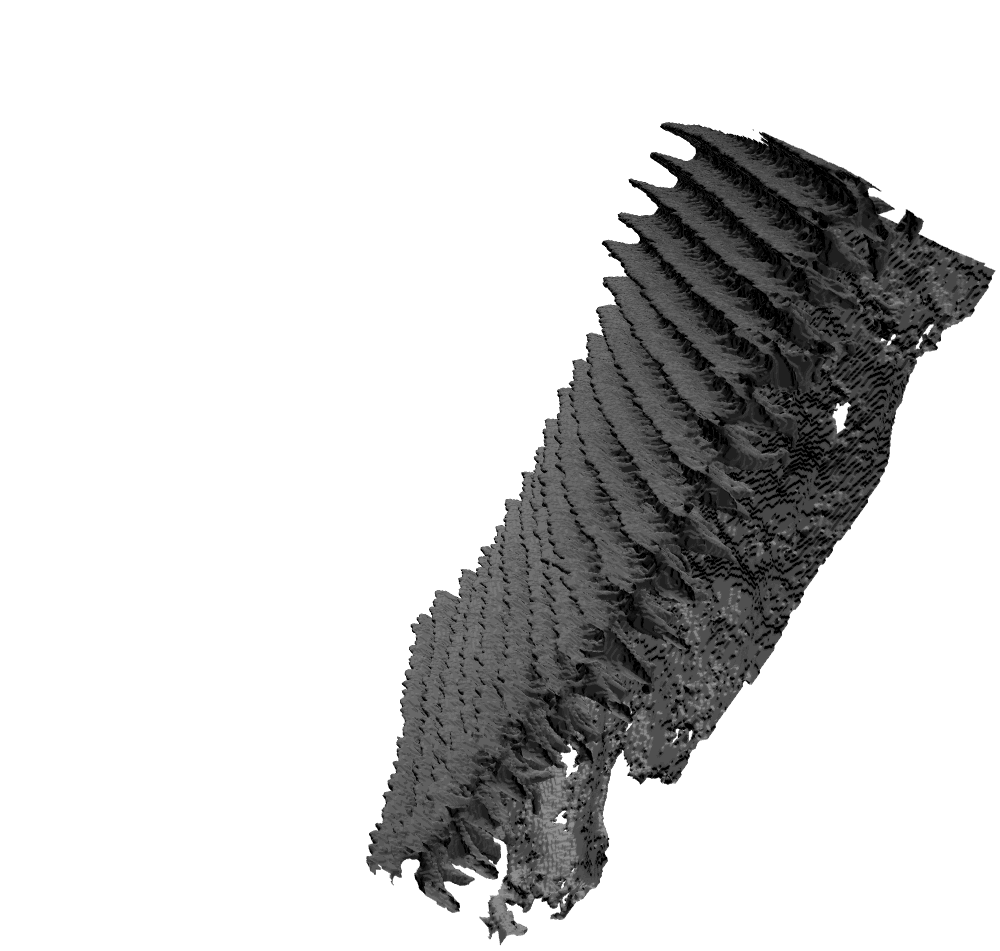

Supplement: Supplementary file 6 — Supplementary Movie 2 [file 41467_2024_48060_MOESM6_ESM.gif]
